# Higher-order temporal network prediction and interpretation

**DOI:** 10.1371/journal.pone.0323753

**Published:** 2025-05-29

**Authors:** H.A. (Bart) Peters, Alberto Ceria, Huijuan Wang

**Affiliations:** 1 Delft University of Technology, Delft, The Netherlands; 2 Leiden Institute of Advanced Computer Science (LIACS), Leiden University, Leiden, The Netherlands; PLOS ONE, UNITED KINGDOM OF GREAT BRITAIN AND NORTHERN IRELAND

## Abstract

A social interaction (so-called higher-order event/interaction) can be regarded as the activation of a hyperlink among the corresponding individuals. Social interactions can be, thus, represented as higher-order temporal networks that record the higher-order events occurring at each time step over time. The prediction of higher-order interactions is usually overlooked in traditional temporal network prediction methods, where a higher-order interaction is regarded as a set of pairwise interactions. The prediction of future higher-order interactions is crucial to forecast and mitigate the spread of information, epidemics and opinion on higher-order social contact networks. In this paper, we propose novel memory-based models for higher-order temporal network prediction. By using these models, we aim to predict the higher-order temporal network one time step ahead, based on the network observed in the past. Importantly, we also intend to understand what network properties and which types of previous interactions enable the prediction. The design and performance analysis of these models is supported by our analysis of the memory property of networks, e.g., similarity of the network and activity of a hyperlink over time, respectively. Our models assume that a target hyperlink’s future activity (active or not) depends on the past activity of the target link and of all or selected types of hyperlinks that overlap with the target. We then compare the performance of our models with three baseline models, which are an activity driven model, a probabilistic group-change model and a pairwise temporal network prediction method. In eight real-world networks, we find that both our models consistently outperform the baselines. Moreover, the refined model, which only uses a subset of all types of overlapping hyperlinks, tends to perform the best. Our models also reveal how past interactions of the target hyperlink and different types of hyperlinks that overlap with the target contribute to the prediction of the target’s future activity.

## Introduction

Temporal networks have been used to represent complex systems with time-varying network topology, where a link between two nodes is activated only when the node pair interacts [1–3]. This classic temporal network representation assumes interactions to be pairwise. While social contacts/interactions have been mostly studied in pairwise temporal networks, they could be beyond pairwise, as individuals may interact in groups [4–6]. For example, a collaboration in a scientific paper may engage more than two authors [7]. Such group interactions, involving an arbitrary number of nodes, are called higher-order interactions or events. Social contacts are thus better represented by higher-order temporal networks, consisting of higher-order interactions, i.e., the activations of hyperlinks (groups of nodes) at specific times.

The classic temporal network prediction problem aims to predict pairwise contacts, one time step ahead, based on the network observed in the past. This problem has applications in a variety of research fields. Predicting a temporal network in the future enables better forecast and mitigation of the spread of epidemics or misinformation on the network, for example. This prediction problem is also equivalent to problems in recommender systems, such as predicting which user will purchase which product, or which individuals will become acquaintances at the next time step [8,9].

Methods have been proposed for pairwise temporal network prediction. Some rely on network embeddings, where nodes are represented as points in a low dimensional embedding space. Within this space, connected nodes are then supposed to be close [10]. Alternatively, deep learning methods have been proposed [11]. Examples are adversarial networks [12] or LSTM methods [13]. The downside of deep learning methods, however, is that they are at the expense of high computational costs, and are limited in providing insights regarding which network mechanisms enable the network prediction. Additionally, other methods have been proposed to predict whether a set of nodes will have at least one group interaction in the future [14–16] and when the first group interaction among these nodes will occur [17]. Lastly, in the design of their network-based prediction method for pairwise temporal networks, Zou *et al*. [18] used the observation of time-decaying memory in social contact networks. Here, this memory property refers to snapshots of the pairwise temporal network sharing more similarities if they are closer in time.

While the forecast of hyperlink activity is usually overlooked in these traditional prediction methods, there have been studies on memory in higher-order temporal networks. Recent results have shown that, in physical contact networks, the activity of hyperlinks with different orders, overlapping in component nodes, seems temporally correlated [19,20]. Besides that, Gallo *et al*. [21] observed correlation between the number of order *d* events in which a node pair participates together at two times, respectively, with a given time delay. In addition, correlation has been observed in the engagement of a node pair in events of different orders over time as well. They also proposed a mechanistic network model that reproduces the general trend of the correlation as a function of the time delay. However, how to calibrate the large number of parameters of this stochastic model for a network prediction task is still an open question. Hence, this model cannot be used for network prediction at this moment. Lastly, Iacopini *et al*. [22] discovered that the probability for a node to change its group to interact with depends on its activity in history, e.g., how long it has interacted with its current group. Driven by this observation, a network model capturing group change dynamics has been proposed to reproduce the observed phenomena.

In this paper, our aim is to predict higher-order interactions, one time step ahead, based on the higher-order temporal network observed in the past for a duration *L*. Motivated by the major insights into hypergraph memory [19–22] and the time-decaying memory found in pairwise temporal networks [18], we will first explore the memory property, or similarity over time, of higher-order temporal networks. In doing so, our intent is to understand which network properties and types of previous interactions may enable the prediction. Specifically, we study systematically to what extent a network’s topology, the activity of a hyperlink and the activity between neighboring hyperlinks, respectively, remains similar over time. After this analysis, we propose a memory-based model to solve the prediction problem utilizing the memory property observed. This model is a generalization to higher-order of the pairwise model proposed in [18].

Our model assumes that the activity (interacting or not) of a hyperlink (or group) at the next time step is influenced by the past activity of this target hyperlink and all neighboring hyperlinks. The time-decaying memory observed is then incorporated in our model through a universal exponential decay, which ensures that the activity of recent events is more influential than the activity of older events. In the prediction problem, we assume that the groups, each of which interacts at least once in the network (in the past or future), as well as the total number of events per group size (order) at the prediction time step, are known. These assumptions aim to simplify the problem. In addition, the total number of interactions of each order could be influenced by factors such as weather and holidays, other than the network observed in the past. This assumption means that group friendship is known, and we aim to predict which groups with group friendship interact at the prediction step.

Using this general model, we unravel the contribution of the activity of each type of neighboring hyperlink when forecasting a target hyperlink’s future activity. This, together with the memory property observed between neighboring hyperlinks, motivates us to propose a refinement upon the general model. This refined model utilizes the activity of more relevant types of neighboring hyperlinks, which are sub-hyperlinks (sub-groups of the target group), super-hyperlinks (super-groups in which the target group is included), and the target hyperlink itself.

We also propose three baseline models as benchmarks for our prediction models. First, we consider the higher-order activity-driven model as proposed in [23]. In this model, the probability for an order *d* interaction to involve a node is proportional to the percentage of order *d* events that this node engages in the whole network. While Di Gaetano *et al*. [23] used this model to construct higher-order networks and study their structural and topological properties, we will use it for higher-order temporal network prediction. As a second baseline we will apply the group-change model [22] that captures group change dynamics for network prediction purpose. Lastly, we will use a self-driven pairwise temporal network prediction method [18]: it considers the higher-order temporal network observed in the past as a pairwise temporal network, then predicts the pairwise temporal network in the next time step, and ultimately deduces higher-order interactions from the predicted pairwise interactions at the same prediction time step.

In the end, we find that both our models generally perform better than the baselines, as evaluated in eight real-world physical contact networks. Moreover, the refined model outperforms the general model for the prediction of events of order 2 and 3. Lastly, we find that the past activity of the target group is the most important factor in the prediction, followed by the activity of the target group’s super- and sub-groups.

This paper is an extension of our previous work [24], which considered only the refined model and one baseline model without the systematic analysis of the memory property nor the general model to motivate the design of the refined model.

## Network representation

Before stating the definition of a higher-order temporal network, we will start with the definition of the pairwise case. A pairwise temporal network *G* measured at discrete times can be represented as a sequence of network snapshots $G=\{G_{1},G_{2},...,G_{T}\}$, where *T* is the duration of the observation window, $G_{t}=(V,E_{t})$ is the snapshot at time step *t*. Here V and *E*_*t*_ are the set of nodes and interactions, respectively. If nodes *a* and *b* have a contact at time step *t*, then $(a,b)\in E_{t}$. Here, we assume that all snapshots share the same set of nodes V. The set of links in the time-aggregated network is defined as $E={⋃}_{t=1}^{t=T}E_{t}$. In this aggregated network, a pair of nodes is connected with a link if at least one contact occurs between them in the temporal network. The temporal connection or activity of link *i* over time can be represented by a *T*-dimensional vector *x*_*i*_. The elements of this vector are given by *x*_*i*_(*t*), where t∈[1,T]. We have *x*_*i*_(*t*) = 1 when link *i* has a contact at time step *t*, and *x*_*i*_(*t*) = 0 if no contact occurs at *t*. A temporal network can thus be equivalently represented by its aggregated network, where each link *i* is further associated with its activity time series *x*_*i*_.

Social interactions, which may involve more than two individuals, can be more accurately represented by the activation/interaction of hyperlinks in a higher-order temporal network *H*. This network is a sequence of network snapshots $H=\{H_{1},...,H_{T}\}$, where $H_{t}=(V,{ℰ}_{t})$ is the snapshot at time step *t*. Here, V represents the set of nodes shared by all snapshots and ${ℰ}_{t}$ is the set of hyperlinks that are activated at time step *t*. The activation of a hyperlink $\left(u_{1},...,u_{d}\right)$ then corresponds to a group interaction among nodes *u*_1_, ..., *u*_*d*_. The hyperlink $\left(u_{1},...,u_{d}\right)$ active at time step *t* is called an event or interaction of size *d*. The set of hyperlinks in the higher-order time-aggregated network is defined as $ℰ={⋃}_{t=1}^{t=T}{ℰ}_{t}$. A hyperlink belongs to ℰ if it is activated at least once in the temporal network. A higher-order temporal network can thus be equivalently represented by its higher-order aggregated network, where each hyperlink *i* is further associated with its activity time series *x*_*i*_.

The previous activity of neighboring hyperlinks may contribute to the prediction of a target hyperlink’s activity in the future. We therefore define different types of neighboring hyperlinks for a given target hyperlink. For an arbitrary target hyperlink *h*, an arbitrary neighboring hyperlink $h^{\prime }$ is called a type $\phi =(dd^{\prime }o)$-neighboring hyperlink of *h*. Here, *d* and $d^{\prime }$ are the sizes of hyperlinks *h* and $h^{\prime }$, respectively, while *o* is the number of nodes shared between both hyperlinks. It holds that $1\leq o\leq \min(d,d^{\prime })$. The unique $\phi =(dd^{\prime }o)$-neighboring hyperlink when $d=d^{\prime }=o$ refers to the target hyperlink *h* itself. Two other particular classes of ϕ-neighboring hyperlinks are the so-called sub- and super-hyperlinks. We say that $h^{\prime }$ is a sub-hyperlink of *h* if $h^{\prime }\subset h$, i.e., if $h^{\prime }$ is included in *h*. At the same time, we call *h* a super-hyperlink of $h^{\prime }$. Given an arbitrary hyperlink of order *d*, all types ϕ of neighboring hyperlinks are denoted as set $\Phi^{d}$. Table 1 displays all possible types ϕ neighboring hyperlinks up to order 4 for an order *d* target link where d∈[2,3,4].

**Table 1:** All possible types ϕ of neighboring hyperlinks upto order 4 for a target hyperlink of order d∈[2,3,4].

order *d*	ϕ			
	target	sub-hyperlinks	sup-hyperlinks	other
2	222	-	232, 242	221, 231, 241
3	333	322	343	321, 331, 332, 341, 342
4	444	422, 433	-	421, 431, 432, 441, 442, 443

## Datasets

To design and evaluate our network prediction methods, we consider eight empirical physical contact networks from the SocioPatterns project. They are publicly available collections of face-to-face interactions in social contexts such as study places (Highschool2012 [25], Highschool2013 [26], Primaryschool [27]), conferences (SFHH Conference [28], Hypertext2009 [29]), workplaces (Hospital [30], Workplace [28]) or an art gallery (Science Gallery [29]). These events are recorded as a set of pairwise interactions, provided that the distance between people is smaller than 2 meters. Based on these pairwise contacts, group interactions are then deduced by promoting every fully-connected clique of (d2) contacts to an event of size *d*. Since a clique of order *d* contains all its sub-cliques of orders $d^{\prime }<d$, only the maximal clique is promoted to a higher-order event. This method has been used in [19–22], where sub-cliques are ignored as well.

The datasets are further preprocessed in a similar way as done in [20] and [31]. We start by removing nodes that are not connected to the largest connected component in the pairwise time-aggregated network. After that, we neglect long periods of inactivity in the network. Such periods usually correspond to nights and weekends, and are therefore recognized as outliers. We then end up with eight preprocessed datasets, whose total number of events of each order is shown in Table 2. Because of the negligible small number of events of orders higher than 4 in most networks, we will consider target hyperlinks as well as their neighboring hyperlinks up to order 4 in our network analysis and the two prediction models we will propose.

**Table 2:** Number of events of every order for every dataset after preprocessing.

Dataset	Order 2	Order 3	Order 4	Order 5	Order 6
Science Gallery	12770	1421	77	7	0
Hospital	25487	2265	81	2	0
Highschool 2012	40671	1339	91	4	0
Highschool 2013	163973	7475	576	7	0
Primaryschool	97132	9262	471	12	0
Workplace	71529	2277	14	0	0
Hypertext 2009	18120	874	31	12	4
SFHH Conference	48175	5057	617	457	199

**Fig 1:**
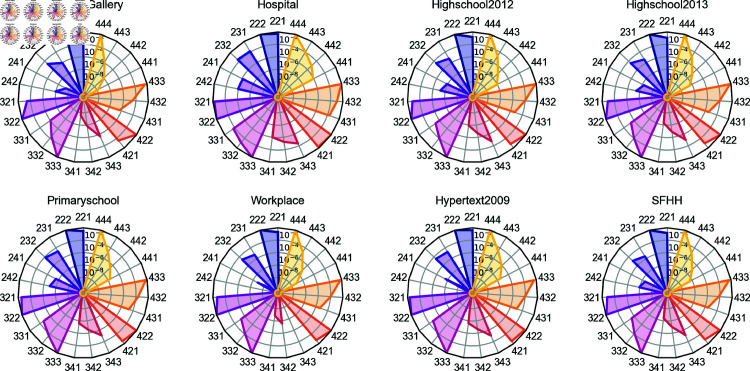
Average number of type $\phi =(dd^{\prime }o)$ neighboring hyperlinks per target hyperlink of order d, normalized by (do)(N−dd′−o), where N is the number of nodes in each network.

Fig 1 displays the average number of neighboring hyperlinks of each type $\phi \in \Phi^{d}$, per target hyperlink of a given order *d*, in each higher-order temporal network. It has been normalized by the number of possible $\phi =(dd^{\prime }o)$-neighboring hyperlinks of an order *d* target hyperlink given the number of nodes *N* in the network, i.e., (do)(N−dd′−o). We find that, on average, an order *d* hyperlink tends to connect to more type $\phi_{1}$-neighbors than $\phi_{2}$-neighbors, if $\phi_{1}=(dd^{\prime }o_{1})$, $\phi_{2}=(dd^{\prime }o_{2})$ and $o_{1}>o_{2}$. In other words, the probability for an order $d^{\prime }$ hyperlink to connect to an order *d* target hyperlink is higher if they overlap more in component nodes. In the next section, we will explore the correlation between a target’s activity and the activity of each type ϕ of neighboring hyperlink, either overlapping significantly with the target or not.

## Network memory property

In this section, we aim to explore the memory property of higher-order temporal networks, which will be utilized in the model design to predict higher-order events. We will first examine the Jaccard similarity between network snapshots and the auto-correlation of a hyperlink’s activity time series. Both similarity measures will then be studied as a function of time lag between snapshots or time series, respectively. They will give us insights in the memory/similarity in network topology and activity of each hyperlink over time, respectively. After that, we will explore if the activity of a target hyperlink/group is correlated with the activity of each type of neighboring hyperlink. We will also examine this correlation as a function of time lag, thereby studying whether certain types of neighboring hyperlinks possess memory of the target link in activity.

### Jaccard similarity between network snapshots

Firstly, we explore the similarity of a higher-order temporal network at order *d*, which contains only order *d* events in the network, over time. We examine the Jaccard similarity $J_{d}(\Delta )$ of a higher-order temporal network at order *d* at two different time steps, separated by a time lag Δ, where d∈[2,3,4]. The Jaccard similarity measures how similar two given sets are by taking the ratio of the size of their intersection set over the size of their union set. In our case, we compute the Jaccard similarity

$$\frac{\left|{ℰ}_{t}^{d}\cap {ℰ}_{t+\Delta }^{d}\right|}{\left|{ℰ}_{t}^{d}\cup {ℰ}_{t+\Delta }^{d}\right|}$$ (1)

between ${ℰ}_{t}^{d}$, the set of *d*-hyperlinks (hyperlinks of order *d*) active at time step *t*, and ${ℰ}_{t+\Delta }^{d}$. Its average over time *t* is referred to as the Jaccard similarity $J_{d}(\Delta )$ of a higher-order temporal network at order *d* with a time lag Δ. A large $J_{d}(\Delta )$ implies a large overlap/similarity between two snapshots of a temporal network at order *d*, separated by a time lag Δ.

**Fig 2:**
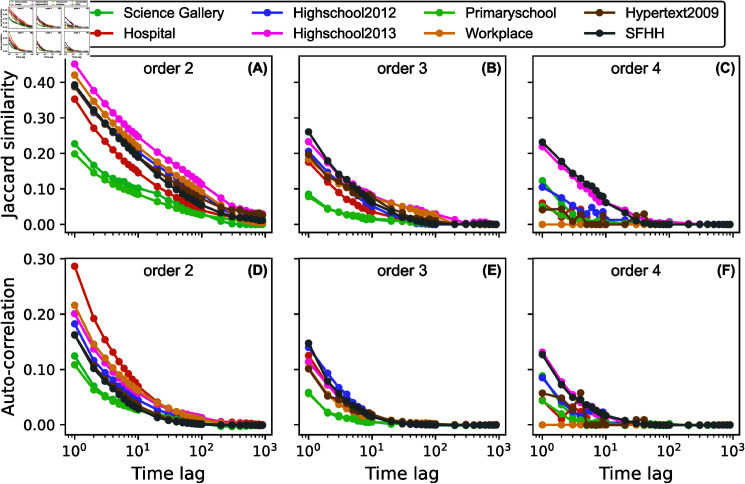
Jaccard similarities (A- C) and average auto-correlations (D- F) of eight real-world physical contact networks for events of order d∈[2,3,4] as a function of time lag.

Figs 2A-C display the Jaccard similarity of each real-world higher-order temporal network at each order d∈[2,3,4]. We observe that, for all datasets, the similarity decays as the time lag increases. Besides that, we find that as the order increases, the slope of the time-decaying curves increases accordingly.

### Auto-correlation of the activity of a hyperlink

Secondly, we look at the average auto-correlation of the activity time series of a hyperlink in a higher-order aggregated network. The auto-correlation of a time series is the Pearson correlation between the given time series and its lagged version. Concretely, the auto-correlation of the activity of a hyperlink *i* is the Pearson correlation coefficient $R_{i,i}(\Delta )$ between $\{x_{i}(t){\}}_{t=1,2,...,T-\Delta }$ and $\{x_{i}(t){\}}_{t=\Delta +1,\Delta +2,...,T}$. Figs 2D-F show the average auto-correlation over all hyperlinks of order *d*, where d∈[2,3,4]. Similar to the Jaccard similarities displayed in Figs 2A-C, we observe time-decaying memory or auto-correlation for the activity of each hyperlink of any order *d*, and the decay is faster if the order is larger.

Our findings of the auto-correlation observed at each hyperlink, together with the Jaccard similarity observed at network level for each order, indicate that the previous activity of a hyperlink could be useful for the prediction of the future activity of this hyperlink. These conclusions are consistent with the findings of Gallo *et al*. [21]. In their paper, they examined the correlation between the number of order *d* events in which a node pair participates together at two times, respectively, with a given time delay. This correlation is thus observed at node pair level, by aggregating the involvement of a node pair in different order *d* events. This method has also been generalized to study the correlation of the engagement of a node pair in events of different orders.

Differently, we examine correlation at a more granular level, namely the single hyperlink level, without decomposing hyperlink activities to their corresponding component node pairs. The correlation observed at hyperlink level could directly motivate the design as well as parameter choice of the higher-order temporal network prediction model that we will propose in section ‘General model’.

### Correlation between neighboring hyperlinks in activity

Fig 1 shows the average number of each type ϕ-neighboring hyperlinks possessed by each target hyperlink in each dataset. We wonder whether a target hyperlink shares similarity in activity with its neighboring hyperlinks, such that we could use the previous activity of these neighbors to predict the future activity of a given target link. In order to study this, we first compute, for each target hyperlink *i*, the Pearson correlation coefficient $R_{i,\phi }(\Delta )$ between the activity of the target *i* and the sum of the activity time series of all its neighboring hyperlinks of type ϕ, with a time lag Δ. We analyze this correlation to inspire the design of our network prediction models which use the aggregated activity of ϕ-neighboring hyperlinks in the past to predict the future activity of a target link.

**Fig 3:**
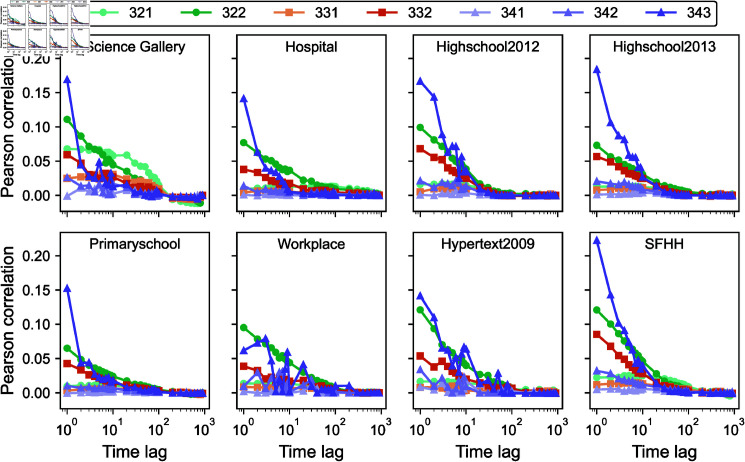
Average Pearson correlation coefficient $R_{i,\phi }(\Delta )$ in activity between an order 3 target hyperlink and ϕ-neighboring hyperlinks in eight real-world physical contact networks as a function of time lag Δ.

We take order 3 target hyperlinks as an example. Fig 3 shows the average Pearson correlation coefficient $R_{i,\phi }(\Delta )$ of order 3 target hyperlinks that have at least one ϕ-neighbor. We find that the correlation is more evident with type ϕ=322 and ϕ=343 neighbors, which are sub- and super-hyperlinks of the target, respectively, and with ϕ=332-neighbors. The correlation with these three types of neighbors is also decaying with Δ, and we observe from Fig 1 that these neighbors are relatively more present than other neighbors of the same order. Besides that, we observe that the correlation in activity with all other types of neighbors is negligibly small.

The average Pearson correlation coefficients $R_{i,\phi }(\Delta )$, between an order 2 or 4 target hyperlink and a ϕ-neighboring hyperlinks, are displayed as a function of time lag Δ in S1 Fig and S2 Fig in the ‘Supporting information’, respectively. The same has been observed that the correlation in activity between a target link with a sub- or super-hyperlink tends to be stronger, and that this correlation/memory is time-decaying. Moreover, among the super- and sub-hyperlinks of a target hyperlink of any order, those that overlap more with the target in nodes are more strongly correlated with the target link.

## Models

In this section we will describe our prediction models and motivate their design. First, we will discuss three baseline models. After that we will discuss our general prediction model, designed based on the memory-property observed in the previous section. Ultimately, we will propose a refinement upon this general model, including only a select group of neighboring hyperlinks in the prediction rather than all possible neighbors as used in the general model.

### Baseline models

#### Higher-order activity-driven model.

We first consider a higher-order activity-driven model as proposed in [23] for higher-order temporal network prediction. In this model, each node *i* in a network is given an activity potential ${𝐚}_{i}=(a_{i}^{\left(2\right)},a_{i}^{\left(3\right)},a_{i}^{\left(4\right)})$, where $a_{i}^{\left(d\right)}$ represents the probability that a random interaction of order *d* at a given time step involves node *i*. Here, $a_{i}^{\left(d\right)}$ is determined by the number of group interactions of order *d* involving node *i* normalized by the total number of order *d* interactions in the network over [1,*T*]. In our targeting prediction problem, it is assumed that the higher-order aggregated network is known, instead of the activity potential of each node.

This model generates a higher-order temporal network as follows. At each prediction time step, we assume the number of order *d* interactions is known, where d∈[2,3,4], which is the same as for the targeting prediction problem. Each order *d* interaction at the prediction step is generated independently by selecting a single node *i* with a probability proportional to its activity potential $a_{i}^{\left(d\right)}$ first. After that, a hyperlink is randomly selected from the set of all possible *d*-hyperlinks that involve node *i* throughout the network. The set of predicted hyperlinks then serves as the prediction made by this activity-driven model.

#### Group-change model.

We further consider the group-change model proposed in [22] as a benchmark model. In this model, each node in the network, either isolated or active in a group interaction, decides if and how it changes to a different group to interact at every prediction time step. It has been observed that the probability for a node to change the group of interaction decreases with the time spent consecutively within that group, in both pairwise [32,33] and higher-order [19,22] interactions. Therefore, the probability of node *i* to change its current group of size *d* is defined as

$$p_{d}(\tau )=\frac{b_{d}}{(\tau^{\beta }+1)/N}.$$ (2)

Here, τ is the number of time steps that node *i* has already spent previously and consecutively in its current group, *N* is the total number of nodes in the network, and *b*_*d*_ and β are derived from the higher-order temporal network *H* through [1,*T*]. Specifically, the distribution of the probability for a random node to change its group at a random time step is derived from *H* and β is the exponent of the power-law fit of this distribution. Similarly, the distribution of the probability for a node residing in an order *d* group to change its group could be derived from *H*, and the intercept is *b*_*d*_. Instead of using *b*_*d*_ where d∈[1,4], we fit the set of coefficients *b*_*d*_ using

$$b_{d}=\frac{d_{0}}{1+e^{-\alpha (d-1)}},$$ (3)

and use the *b*_*d*_ of the fitted function to compute the probability $p_{d}(\tau )$ for a node to change its current group of size *d*. If a similar fitting curve can be observed in diverse networks, we may estimate *b*_*d*_ without knowing the higher-order temporal network *H*. However, the fitting curve 3 for the real-world networks considered in this paper slightly differs from that derived from the networks used in [22]. Hence, the network *H* is still needed to derive the input parameters β and *b*_*d*_ of this group change model.

Secondly, the model selects the new group to interact if the node decides to switch groups. If we denote the current group of node *i* at time *t* by $\sigma_{i}^{t}\in {𝒦}^{t}$, where ${𝒦}^{t}$ is the set of active groups at *t*, then we denote the new group after a group change by $\omega \in \Omega_{t}=\{{𝒦}^{t}\backslash \sigma_{i}^{t}$ ∪ ∅}. In fact, we include multiple empty sets, the multiplicity governed by a control parameter ϵ, which we consider to be ϵ=100 for our networks, thereby allowing the node to become isolated. Node *i* then joins a group $\omega \in \Omega_{t}$ with a probability proportional to the fraction of nodes in ω that node *i* either has already encountered in previous group interactions in the time interval [*t*–*L*,*t*] or possibly will encounter at *t* + 1. In the case of a group change, group $\sigma_{i}^{t}$ will be disbanded. At the prediction time step, this process is repeated for all individual nodes, and the resulting set of groups is considered to be predicted. This model does not use the total number of interactions for each order in the prediction.

#### Self-driven model.

As a third baseline we propose a model that utilizes the following pairwise network prediction model, called the self-driven model, proposed in [18]. The self-driven model is a memory-based model that predicts a pairwise link’s activity at the next time step, based on its past activity, assuming the pairwise aggregated network over [1,*T*] is known. This estimates the tendency *w*_*i*_(*t* + 1) for each link *i* to be active at time *t* + 1 as

$$w_{i}(t+1)=\sum_{k=t-L+1}^{k=t}x_{i}(k)e^{-\tau (t-k)}.$$ (4)

Here, *t*  +  1 is the prediction time step, *L* is the length of the past observation used for the prediction, τ is the exponential decay factor and *x*_*i*_(*k*) is the activation state of link *i* at time *k*. So we have *x*_*i*_(*k*) = 1 if the link *i* is active at time *k* and *x*_*i*_(*k*) = 0 otherwise. The exponential function in Eq 4 captures the memory of the network, thereby ensuring that recent events have more influence than older events. We compute the activation tendency for each link in the pairwise aggregated network in the prediction step *t*  +  1. Given the pairwise aggregated network and the number of pairwise contacts occurring at *t*  +  1, the same number of pairwise links in the aggregated network with the highest activation tendency at *t*  +  1 will then be predicted to be active.

The self-driven model is adapted for higher-order temporal network prediction as follows. It is assumed that the higher-order aggregated network throughout the time window [1,*T*] is known, and its corresponding pairwise aggregated network, that regards each hyperlink in the higher-order aggregated network as a set of pairwise links between every two component nodes, will be used by the model. First, we consider the higher-order temporal network observed for the past *L* time steps as a pairwise temporal network. After that, we apply Eq 4 to predict pairwise interactions at the prediction step. From those interactions, we then deduce higher-order interactions using the same method that promotes pairwise interactions that form a clique to a higher-order event, as described in section ‘Datasets’. This set of higher-order interactions is considered the prediction made by the self-driven model.

### General model

Beyond pairwise temporal networks, we have also observed time-decaying memory in higher-order temporal networks at each group order. This motivates us to generalize the self-driven model for higher-order temporal network prediction. The essence of this general model is that the future activity of a hyperlink should be dependent on its past activity. Furthermore, Fig 3 showed us that the activity of a target hyperlink shares similarity with the activity of its neighboring hyperlinks. Hence, the activity of a hyperlink is possibly dependent on the past activity of its neighboring hyperlinks. Finally, recent events should have more influence than older events, based on the time-decaying memory observed in the auto-correlation of the activity of each hyperlink and the correlation in activity between a target and its neighboring hyperlinks. Therefore, we propose that the activation tendency of a hyperlink *i* of order *d* at a prediction time step *t* + 1 is given by

$$w_{i}(t+1)=\sum_{\phi \in \Phi^{d}}c_{\phi }y_{i}^{\phi }(t)+c_{d},$$ (5)

where *c*_*d*_ is a constant, called the intercept, and

$$y_{i}^{\phi }(t)=\sum_{k=t-L+1}^{k=t}\;\sum_{j\in S_{i}^{\phi }}x_{j}(k)e^{-\tau (t-k)}.$$ (6)

In Eq 6, *L* resembles the time span used for prediction, τ is the exponential decay factor, and $S_{i}^{\phi }$ corresponds to set of all type ϕ neighboring hyperlinks of the target *i*, including the target itself. The contribution of each type ϕ of neighboring hyperlinks $y_{i}^{\phi }(t)$, to estimate the activation tendency *w*_*i*_(*t*  +  1) of a target hyperlink *i*, includes the activity of all hyperlinks in the set $S_{i}^{\phi }$, over the observation period of length *L* with an exponential decay over time. For a target hyperlink *i* of order *d*, the contribution of all possible types $\phi \in \Phi^{d}$ of neighboring hyperlinks is taken into account. The activation tendency *w*_*i*_(*t*  +  1) is assumed to be a linear function of the contribution of each type $\phi \in \Phi^{d}$ of neighboring hyperlinks associated with a coefficient $c_{\phi }$, which we assume to be the same for all target hyperlinks of the same order.

All coefficients will be learned via Lasso regression that, for all target hyperlinks of order *d*, minimizes the objective function

$$\min_{C^{d}}\left\{\sum_{i}\sum_{t+1}{\left((x_{i}(t+1)-w_{i}(t+1)\right)}^{2}+\alpha \sum_{c\in C^{d}}|c|\right\}.$$ (7)

Here, *x*_*i*_(*t* + 1) is the true activity of hyperlink *i* at *t* + 1, and ${\text{C}}^{\text{d}}$ is the set consisting of the intercept *c*_*d*_ and all coefficients $c_{\phi }$, where $\phi \in \Phi^{d}$. A unique set of coefficients will be learned for all target hyperlinks with a given order *d*, by considering the true activity of each order *d* hyperlink *i* at every prediction time step t+1∈[L+1,T] and the corresponding activity of this link *i* and its neighboring hyperlinks in the previous *L* time steps to derive the prediction *w*_*i*_(*t* + 1).

We use L1 regularization, which adds a penalty to the sum of the magnitude of coefficients. The parameter α controls the penalty strength. The regularization forces some of the coefficients to be zero and thus leads to models with few non-zero coefficients (relevant features). The optimal α is achieved through a grid search in a set of 200 logarithmically spaced points within [10^−19^, 10].

Using the learned optimal fitting coefficients, the activation tendency is computed for each hyperlink in the higher-order aggregated network, which is given in the prediction problem. Given the number $n_{t+1}^{d}$ of events of each order *d* at the prediction step *t*  +  1, the $n_{t+1}^{d}$ hyperlinks of order *d* with the highest activation tendency at *t*  +  1 are then predicted to be active. Since the hyperlink forecast is performed for each order separately, the prediction of events of a given order does not influence the prediction of another order.

In the context of the aforementioned real-world contact networks, whose number of events of an order higher than 4 is negligible small, we aim to predict the activity of target hyperlinks of order d∈[2,3,4] using the activity of the target hyperlink and neighboring hyperlinks upto order 4 via the general model and refined model, respectively.

### Refined model

Some types of neighboring hyperlinks are possibly more relevant for the prediction than others. If so, better prediction can possibly be achieved via including only more relevant types of neighboring hyperlinks instead of considering all types of neighboring links as in our general model. Therefore, we will explore the optimal coefficients learned for the general model discussed in the previous section, as well as the correlation in activity between neighboring hyperlinks analyzed in section ‘Correlation between neighboring hyperlinks in activity’, to identify the relatively more relevant types of neighboring hyperlinks and propose a refined model.

We consider τ=5 and *L* = 30 as an example for the model design and performance analysis. It has been shown that τ∈[0.5,5] leads to an optimal prediction accuracy for pairwise temporal network prediction [18], thus there is no need to calibrate τ. The choice of *L* could be limited thus driven by the context of real-world prediction problem, e.g., how long of the network in the past can be used for the prediction. A small *L* like *L* = 30 is more feasible in practice and leads to a lower computational complexity. In section ‘Performance analysis of the refined model’, the influence of these two parameters on the prediction quality will be discussed.

The coefficients that weigh the contributions of all possible types of neighboring hyperlinks in estimating the connection tendency of an order 3 target hyperlink, obtained by Lasso regression, are given in Table 3. We notice that the coefficient corresponding to the target hyperlink is generally the largest. This is likely because the auto-correlation, or memory in the activity of a hyperlink itself, as shown in Fig 2, is evident and generally larger than the activity correlation with its ϕ-neighbors as displayed in Fig 3. Furthermore, we observe from Table 3 that the coefficients for sub- and super- hyperlinks tend to be larger than those for other types of neighboring hyperlinks. This is in line with our analysis in section ‘Correlation between neighboring hyperlinks in activity’, where we showed that these neighboring groups have generally a larger correlation with target hyperlinks than other ϕ-neighbors. While these two observations regarding the coefficients also hold for the forecast of 2-events, they are not always true for the coefficients used to predict order 4 events, due to the low number of order 4 hyperlinks. The coefficients for orders 2 and 4 are shown in the ‘Supporting information’ in S1 Table and S2 Table, respectively.

**Table 3:** Coefficients of the generalized model to predict order 3 events, when *L* = 30 and τ=5.

Dataset	$c_{333}$	$c_{321}$	$c_{322}$	$c_{331}$	$c_{332}$	$c_{341}$	$c_{342}$	$c_{343}$	$c_{3}$
Science Gallery	0.15	0.00	0.03	0.00	0.02	0.00	0.01	0.19	0.00
Hospital	0.28	0.00	0.01	0.00	0.00	0.00	0.00	0.16	0.00
Highschool2012	0.35	0.00	0.01	0.00	0.01	0.00	0.00	0.24	0.00
Highschool2013	0.34	0.00	0.01	0.00	0.01	0.00	0.00	0.13	0.00
Primaryschool	0.16	0.00	0.01	0.00	0.01	0.00	0.01	0.17	0.00
Workplace	0.28	0.00	0.01	0.00	0.01	0.00	0.01	0.20	0.00
Hypertext2009	0.35	0.00	0.02	0.00	0.01	0.00	0.00	0.05	0.00
SFHH Conference	0.37	0.00	0.01	0.00	0.01	0.00	0.00	0.15	0.00

Both observations in coefficients when predicting order 2 and 3 events, supported by our network memory analysis, motivate us to consider the following refined model. It is the same as the general model, but includes only the contribution of the target hyperlink itself and the sub- and super- neighboring hyperlinks when estimating the connection tendency *w*_*i*_(*t* + 1) of the target hyperlink. Similarly, the coefficients weighing the contributions of sub- and super-links could be learned via Lasso Regression. Using these optimal coefficients learned from the whole data set over [1,*T*], the connection tendency of each target hyperlink can be computed at any prediction time t+1∈[L+1,T], based on the network observed within [t−L+1,t]. Given the total number of events of each order at *t* + 1, the higher-order temporal network can be predicted by considering hyperlinks with the highest connection tendency at each order to be active.

## Model evaluation

We will now evaluate our models on our eight real-world social contact networks. The performance of all models will be evaluated using the prediction quality, as will be explained in the next subsection. After that we will further examine the performance of the refined model by studying the effect of the model parameters and ϕ-coefficients on its prediction quality.

### Network prediction quality

Using the optimal coefficients, each model predicts the higher-order network, i.e., events of each order, at any prediction time *t*  +  1, based on the network observed within [t−L+1,t]. The network prediction quality at any prediction time *t*  +  1 of a given model for events of an arbitrary order is evaluated via the prediction accuracy. This accuracy corresponds to the ratio between the total number of true positives (correctly predicted events) and the total number of events. The prediction quality of a model for events of a given order is the average accuracy over all possible prediction times t+1∈[L+1,T].

In the case of the self-driven, general and refined model, multiple hyperlinks of a given order *d* may have the same activation tendency at a given prediction step *t*  +  1. Given the actual number of order *d* events at *t*  +  1, the order *d* events predicted are, thus, possibly not unique. To account for this, we consider 100 realizations of order *d* event prediction and consider their average accuracy at each prediction step *t*  +  1. The higher-order activity-driven and group-change models are also probabilistic. Therefore, we consider the average accuracy over 100 iterations of the prediction for the activity-driven model and the average accuracy of 10 iterations of the group-change model, whose computational cost is relatively high.

**Fig 4:**
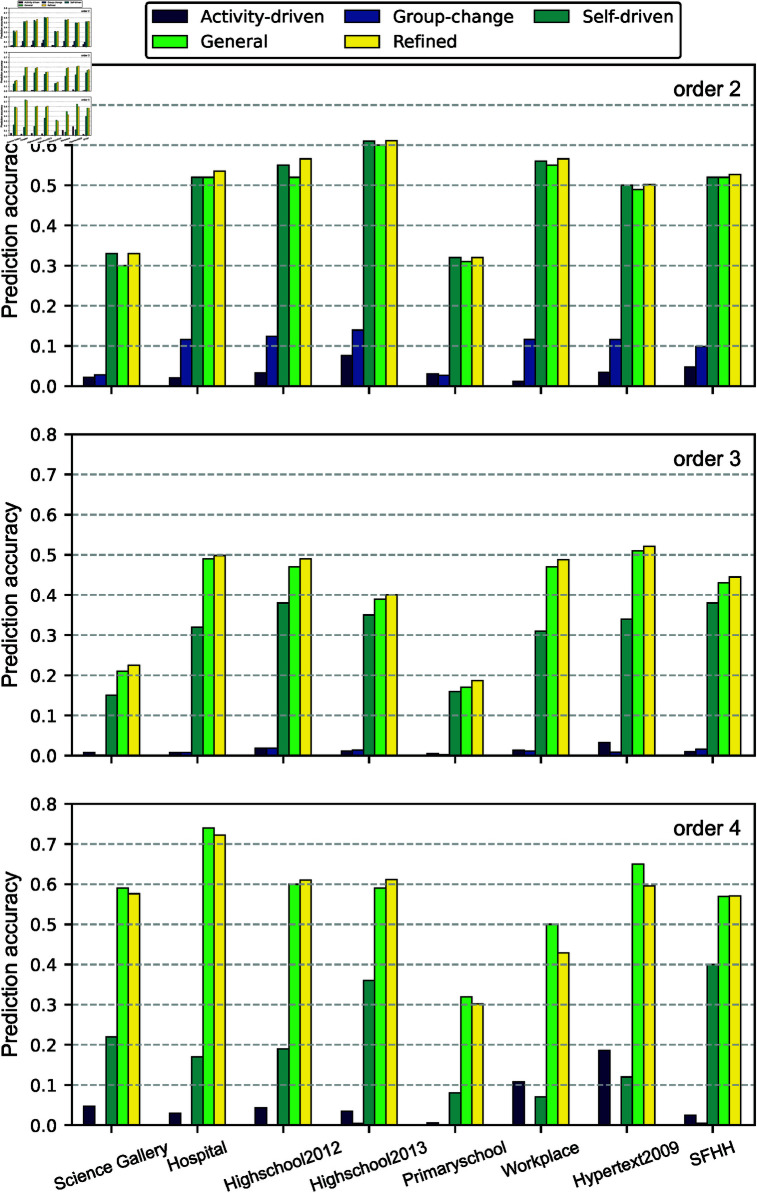
Prediction quality of all models, with L=30, τ=5 and ϵ=100, for 2-, 3-, and 4-events in every dataset.

The prediction quality of all models is displayed in Fig 4. We find that the self-driven, general and refined models evidently outperform the activity-driven and group-change models. In general, the general and refined models that we have proposed tend to perform better than the self-driven baseline model. This out-performance is more evident when predicting events of order 3 and 4.

The refined model performs better than the general model when predicting order 2 and order 3 events. This supports that the refined model captures the contribution of key types of neighboring hyperlinks when predicting the activity of an arbitrary target link. However, the refined and general model perform similarly when predicting order 4 events. This is because the number of order 4 events is small and the design of the refined model is driven by the observation that the sub- and super- hyperlinks contribute more, as reflected in their corresponding coefficients in the general model, in predicting order 2 or 3 events than other types of neighboring hyperlinks, which is less evident when predicting order 4 events. The less evident out-performance of the refined model compared to the self-driven model in predicting order 2 events is because order 2 target links have no sub- and also mostly no super-hyperlinks.

**Fig 5:**
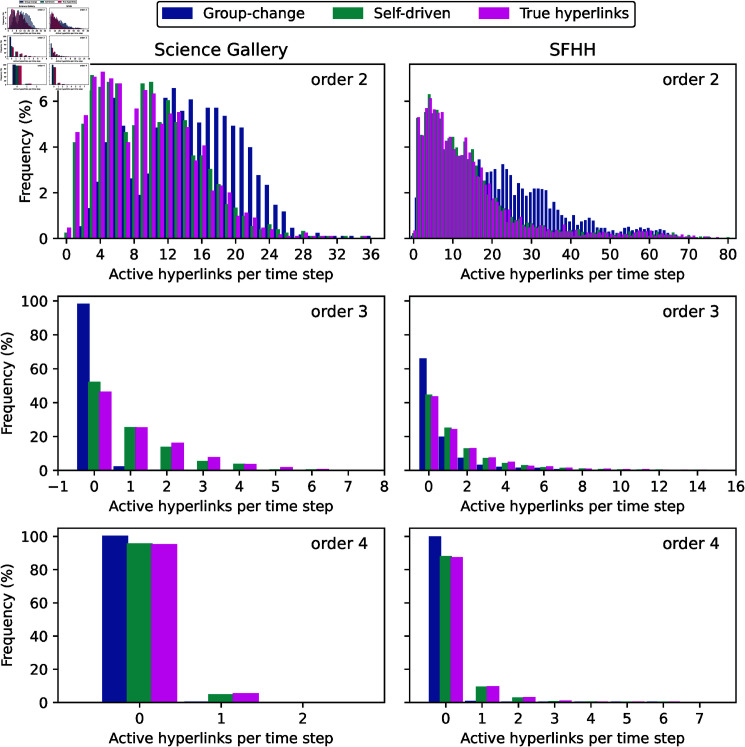
Distributions of the true and predicted number of order d interactions per time step via group-change (L=30, ϵ=100) and self-driven (L=30, τ=5) models in two datasets.

Two baseline models, the self-driven model and the group change model, have not used the the total number of interactions of each order at the prediction step as input as the other models. Hence, we examine the distribution of the number of order *d* interactions predicted by these two models in comparison with that of the ground truth. Fig 5 shows that, for each order *d*, the distribution of the number of interactions predicted by the self-driven model is close to that of the actual number of interactions. This observation does not hold for the group-change model, which seems to consistently generate more events of order 2. The same has been observed for group-change models with a different value of ϵ. Hence, the group-change model fails to predict the total number of events for each order, partially explaining its low prediction quality.

### Performance analysis of the refined model

#### Influence of coefficients $c_{\phi }$.

We previously computed the prediction accuracy for our models by using an optimal set of coefficients, learned via Lasso regression, based on the temporal network observed over the complete time span [1,*T*]. In reality, the network at the prediction step and further in time, thus within [t+1,T], is unknown. Hence, we aim to explore the influence of the coefficients $c_{\phi }$ on the performance of our refined model. This allows us to explore which coefficient ranges tend to lead to close to optimal prediction.

We calculate the prediction accuracy of our refined model, where the coefficient corresponding to the target hyperlink *c*_*ddd*_ = 1, and the coefficients associated with the sub- and super-hyperlinks are chosen from all possible values within [0.0,0.1,...,1.0]×[0.0,0.1,...,1.0]. In this way, we can study the impact of sub- and super-hyperlink coefficients relative to the target hyperlink coefficient *c*_*ddd*_ on the prediction accuracy.

**Fig 6:**
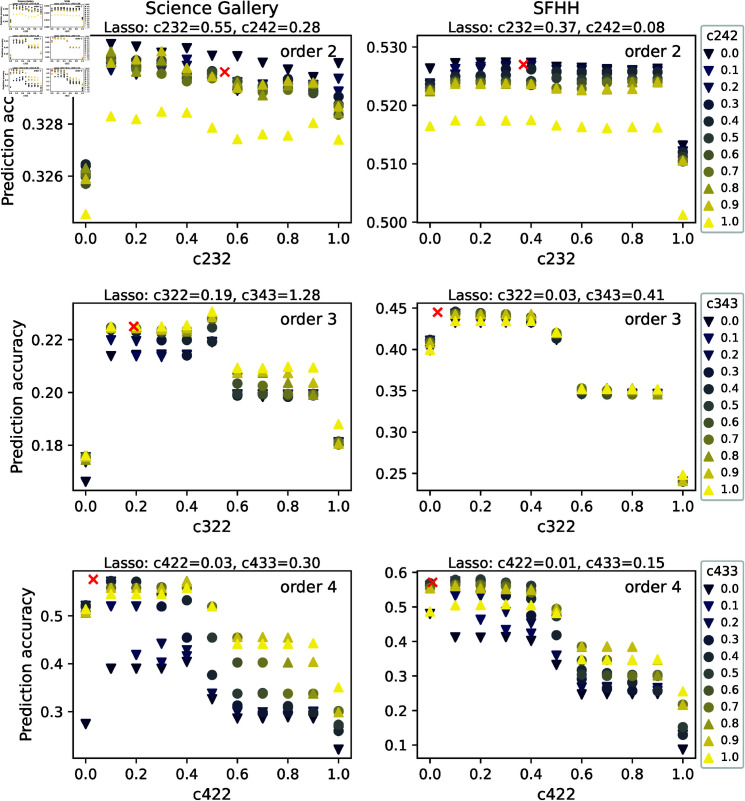
Prediction accuracy of the refined model for events of order d∈[2,3,4] as a function of one coefficient, with the other coefficient fixed, for two datasets.

Fig 6 shows the prediction accuracy as a function of the sub- and super-hyperlink coefficients for every order d∈[2,3,4] in two networks. We take the prediction of order 3 events as an example. We find that that close to optimal prediction accuracy is obtained approximately when $c_{322}\in [0.1,0.4]<c_{333}=1$. This is in line with our finding in section ‘Refined model’, that the past activity of a ϕ=(322)-neighbor contributes less to the prediction of the future activity of an order 3 target link than the past activity of the target itself. The influence of the coefficient *c*_343_ for super-hyperlinks on the prediction quality is in general less than that of the coefficient *c*_322_ for sub-hyperlinks. In general, a $c_{343}\in [0.8,1]$ tends to enable close to optimal prediction accuracy.

We have also added the coefficients of the refined model learned from Lasso regression to Fig 6 as red markers. The parameter analysis in this figure, however, assumes *c*_333_ = 1. Hence, coefficients *c*_322_ and *c*_343_ learned from Lasso are normalized by the learned *c*_333_, and are included in Fig 6 together with their corresponding prediction accuracy. The refined model with the learned coefficients seems to lead to the optimal prediction quality in all networks, but close to optimal in Science Gallery. This is because Lasso Regression aims not solely to optimize the prediction accuracy, but also to minimize the magnitude of coefficients. For the learned coefficient, we observe that $c_{322}<c_{343}$. Consistently, we found the ranges $c_{322}\in [0.1,0.4]$ and $c_{343}\in [0.8,1]$ that tend to lead to close optimal prediction, which also indicates $c_{322}<c_{343}$.

For events of order 2, we find that the prediction quality tends to be optimal when $c_{232},c_{242}\in [0.1,0.4]$. However, their influence on the prediction quality is less evident, as partially shown in the upper panel of Fig 6. For order 4 events (see lower panel of Fig 6), *c*_422_ and *c*_433_ affect the prediction quality evidently. We find that $c_{422}\in [0.1,0.4]$ and $c_{433}\in [0.6,0.9]$ tend to give rise to close to optimal prediction precision. Hence, to achieve the optimal prediction quality, we could choose $c_{422}<c_{433}<c_{444}$ and $c_{322}<c_{343}\leq c_{333}$, thus let a hyperlink that overlaps more with the target hyperlink in nodes to contribute more. This result is complementary to our finding in section ‘Correlation between neighboring hyperlinks in activity’, that among the super- and sub-hyperlinks, those that overlap more with a target hyperlink in nodes are more correlated with the target.

#### Influence of τ and *L.*

It has been shown that τ∈[0.5,5] leads to an optimal prediction accuracy for pairwise temporal network prediction [18]. We are interested if in higher-order temporal networks also a broad range of τ would lead to optimal accuracy.

**Fig 7:**
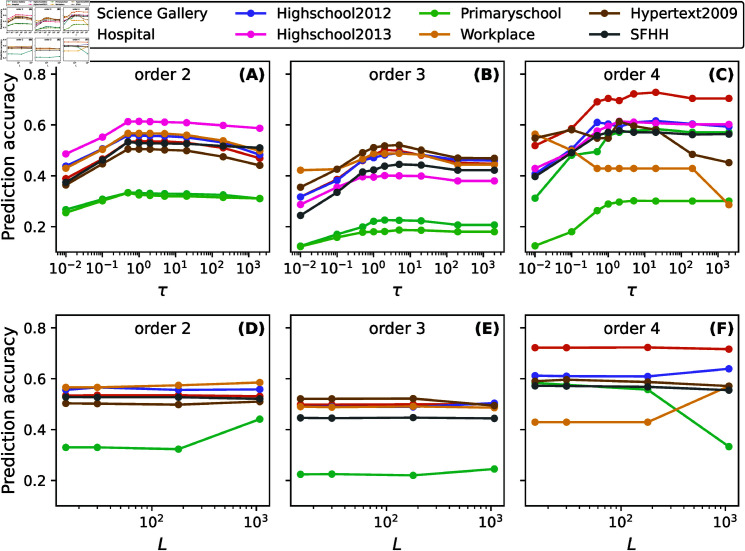
Prediction accuracy of hyperlinks of order d∈[2,3,4] as a function of the exponential decay factor τ (A- C) and observation window L (D- F) in eight and six real-world physical contact networks, respectively.

We now compare the prediction accuracy of the refined model for distinct values of $\tau \in [10^{-2},10^{3}]$ when *L* = 30. Given *L* = 30 and a given τ, the coefficients of the refined model are fitted via Lasso Regression and further used to predict higher-order events. Figs 7A-C show that approximately any τ∈[0.5,5] leads to close to optimal prediction quality in all networks considered, meaning the parameter τ could be chosen arbitrarily within this broad range without the need to be calibrated. Secondly, we generally find a less optimal performance for large values of τ. This is because a large τ results in a fast decay of the exponential in Eq 6, thereby suppressing the contribution of previous events. Lastly, we find that the worst performance is achieved for small values of τ, because when τ is small, previous interactions almost contribute equally in event prediction, independent of when these interactions occur.

As mentioned earlier, the choice of *L* could be driven by the context of real-world prediction problem, e.g., how long of the network in the past is available and can be used for the prediction. A small *L* like *L* = 30 is more feasible in practice and leads to a lower computational complexity. If a temporal network can be observed for long in the past for the prediction of future events, would the choice of *L* influence the prediction quality? In Fig 7D-F we explore the prediction accuracy of events of order d∈[2,4] as a function of L∈[15,30,180,1080] while keeping a fixed τ=5. A value of *L* = 30 means than an observation of a network for 600*s* in the past is used for the prediction, as the duration of each time step is 20 seconds. Hence, the set of *L* considered implies an observation of a duration ranging from 5 minutes to 6 hours to predict the network 20 seconds ahead. Fig 7D-F show that, in six network examples, the prediction quality is relatively stable as *L* varies within L∈[15,30,180]. When *L* is increased to the extreme *L* = 1080, the prediction accuracy could be higher or lower. Hence, L∈[15,30,180] seems to be a good choice, for which data is relatively accessible and the computational complexity is low.

## Conclusion and discussion

In this work, we propose network-based higher-order temporal network prediction models that estimate the activity of each target hyperlink at the next time step, based on the past activity of this target and of its ϕ-neighboring hyperlinks. The general model considers the contribution of neighbors of all types ϕ, motivated by the memory in activity of each hyperlink and between neighboring hyperlinks. The refined model considers only two types of neighboring links, the super- and sub-links, inspired by the coefficients analysis in the general model and the observation that a hyperlink is more strongly correlated with these two types of neighbors than with others.

We discover that the both models consistently outperform three baseline models, i.e., the activity-driven model, the group-change model and the self-driven pairwise network prediction model . The refined model performs better than the general model in the prediction of 2- and 3-events. The optimal coefficients of our models learned via Lasso regression, as well as the correlation analysis between neighboring hyperlinks in activity, unravel that the past activity of the target link itself contributes the most in the prediction, followed by the activity of super- and sub-links. Among these super- and sub-links, those that overlap more with the target link in nodes also contribute more in the prediction.

The identification of the optimal coefficients for the refined model is via the learning from the temporal network in the past as well as in the future, which is unrealistic. Hence, we analyzed the influence of various parameters on the prediction accuracy, and identified ranges of parameters that may lead to close to optimal prediction quality. An interesting future work is to explore whether the refined model, thus the optimal coefficients learned from the network observed in the past, could well predict future events.

In this work, we have focused on higher-order social contact networks that are derived from measurements of pairwise interactions. These networks have the property that a hyperlink and its sub-links are never activated at the same time. It is interesting to explore methods to predict other types of higher-order temporal networks that may not have this property nor the memory property, such as collaboration networks [34]. Moreover, better prediction accuracy can possibly be achieved when a different decay factor τ is considered for each type ϕ of neighbors, because the slope of the time- decaying memory between a target hyperlink and a neighboring hyperlink depends on the type of neighbor. Finally, the baseline group-change model could be further designed to utilize the number of events of each order at the prediction time step as input.

## Supporting information

S1 FigAverage Pearson correlation coefficient for order 2 hyperlinks connecting to neighbors of type $\phi \in \Phi^{2}$ in eight real-world physical contact networks as a function of time lag Δ(TIFF)

S1 TableCoefficients of the general model to predict order 2 events, when L=30 and τ=5.(PDF)

S2 FigAverage Pearson correlation coefficient for order 4 hyperlinks connecting to neighbors of type $\phi \in \Phi^{4}$ in eight real-world physical contact networks as a function of time lag Δ.(TIFF)

S2 TableCoefficients of the general model to predict order 4 events, when L=30 and τ=5.(PDF)
