# Supplementary figures and images for: Higher-order temporal network prediction and interpretation
Source: PLoS One. 2025 May 29;20(5):e0323753. doi: 10.1371/journal.pone.0323753 (PMC12121753; doi:10.1371/journal.pone.0323753)

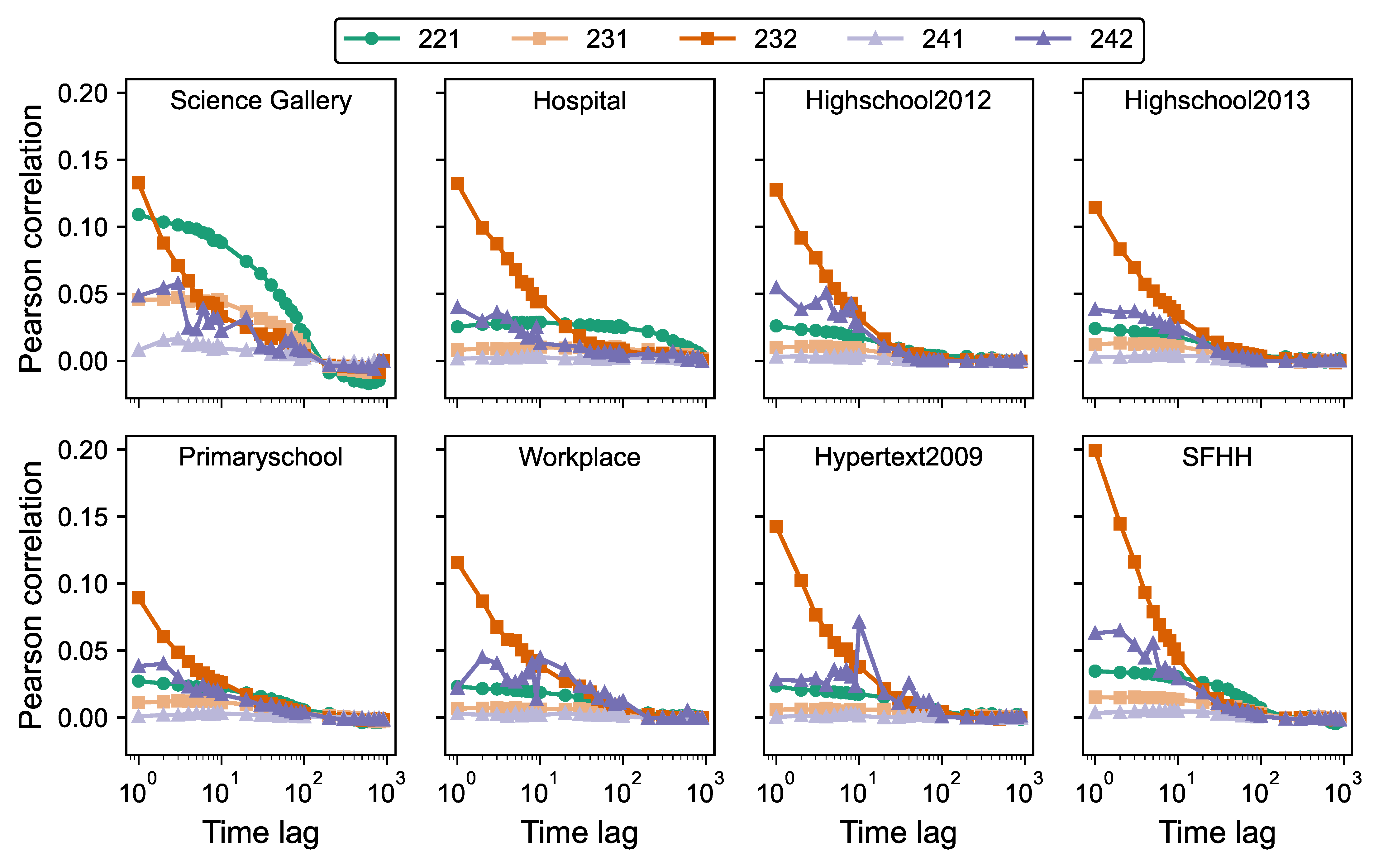

Supplement: S1 Fig — Average Pearson correlation coefficient for order 2 hyperlinks connecting to neighbors of type ϕ∈Φ2 in eight real-world physical contact networks as a function of time lag Δ (TIFF) [file pone.0323753.s001.tif]

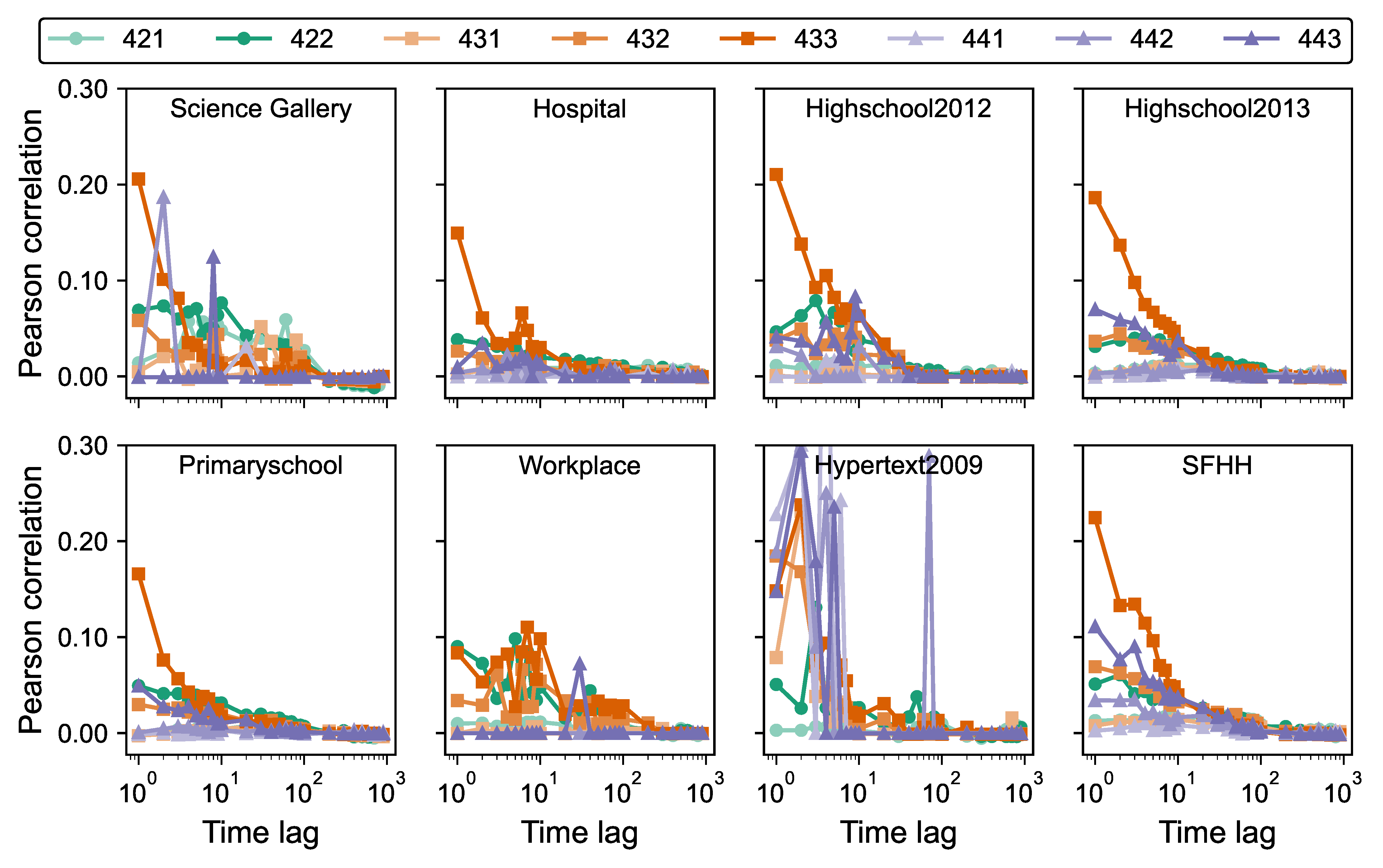

Supplement: S2 Fig — Average Pearson correlation coefficient for order 4 hyperlinks connecting to neighbors of type ϕ∈Φ4 in eight real-world physical contact networks as a function of time lag Δ. (TIFF) [file pone.0323753.s003.tif]
