# Supplementary material for: Higher-order temporal network prediction and interpretation
Source: PLoS One. 2025 May 29;20(5):e0323753. doi: 10.1371/journal.pone.0323753 (PMC12121753; doi:10.1371/journal.pone.0323753)
Supplement: S1 Table — (PDF) [file pone.0323753.s002.pdf]

Higher-order temporal network prediction and interpretation

H.A. (Bart) Peters[1], Alberto Ceria[2], Huijuan Wang[1*]

1 Delft University of Technology, Mekelweg 4, 2628 CD Delft, Netherlands
2 Leiden Institute of Advanced Computer Science (LIACS), Leiden University, Einsteinweg 55, 2333 CC Leiden, The Netherlands

* H.Wang@tudelft.nl

Abstract

A social interaction (so-called higher-order event/interaction) can be regarded as the activation of a hyperlink among the corresponding individuals. Social interactions can be, thus, represented as higher-order temporal networks that record the higher-order events occurring at each time step over time. The prediction of higher-order interactions is usually overlooked in traditional temporal network prediction methods, where a higher-order interaction is regarded as a set of pairwise interactions. The prediction of future higher-order interactions is crucial to forecast and mitigate the spread of information, epidemics and opinion on higher-order social contact networks. In this paper, we propose novel memory-based models for higher-order temporal network prediction. By using these models, we aim to predict the higher-order temporal network one time step ahead, based on the network observed in the past. Importantly, we also intend to understand what network properties and which types of previous interactions enable the prediction. The design and performance analysis of these models is supported by our analysis of the memory property of networks, e.g., similarity of the network and activity of a hyperlink over time, respectively. Our models assume that a target hyperlink's future activity (active or not) depends on the past activity of the target link and of all or selected types of hyperlinks that overlap with the target. We then compare the performance of our models with three baseline models, which are an activity driven model, a probabilistic group-change model and a pairwise temporal network prediction method. In eight real-world networks, we find that both our models consistently outperform the baselines. Moreover, the refined model, which only uses a subset of all types of overlapping hyperlinks, tends to perform the best. Our models also reveal how past interactions of the target hyperlink and different types of hyperlinks that overlap with the target contribute to the prediction of the target's future activity.

Supporting information

S1 Table. Coefficients of the general model to predict order 2 events, when $L = 30$ and $\tau = 5$.

**Dataset**	c_{222}	c_{221}	c_{231}	c_{232}	c_{241}	c_{242}	c_2
Science Gallery	0.31	0.01	0.01	0.17	0.00	0.08	0.00
Hospital	0.50	0.00	0.00	0.22	0.00	0.12	0.00
Highschool2012	0.56	0.00	0.00	0.20	0.01	0.08	0.00
Highschool2013	0.61	0.00	0.00	0.20	0.00	0.06	0.00
Primaryschool	0.31	0.00	0.00	0.17	0.00	0.11	0.00
Workplace	0.55	0.00	0.00	0.23	0.00	0.16	0.00
Hypertext2009	0.48	0.00	0.00	0.23	0.00	0.17	0.00
SFHH Conference	0.52	0.00	0.00	0.19	0.00	0.04	0.00

References

1. Holme P, Saramäki J. Temporal networks. Physics reports. 2012;519(3):97–125.
2. Masuda N, Lambiotte R. A guide to temporal networks. World Scientific; 2016.
3. Holme P. Modern temporal network theory: a colloquium. The European Physical Journal B. 2015;88:1–30.
4. Battiston F, Cencetti G, Iacopini I, Latora V, Lucas M, Patania A, et al. Networks beyond pairwise interactions: structure and dynamics. Physics Reports. 2020;874:1–92.
5. Battiston F, et al. The physics of higher-order interactions in complex systems. Nature Physics. 2021;17(10):1093–1098.
6. Sekara V, Stopczynski A, Lehmann S. Fundamental structures of dynamic social networks. Proceedings of the national academy of sciences. 2016;113(36):9977–9982.
7. Patania A, Petri G, Vaccarino F. The shape of collaborations. EPJ Data Science. 2017;6:1–16.
8. Lü L, Medo M, Yeung CH, Zhang YC, Zhang ZK, Zhou T. Recommender systems. Physics reports. 2012;519(1):1–49.
9. Aleta A, Tuninetti M, Paolotti D, Moreno Y, Starnini M. Link prediction in multiplex networks via triadic closure. Physical Review Research. 2020;2(4):042029.
10. Zhou Y, Pei Y, He Y, Mo J, Wang J, Gao N. Dynamic Graph Link Prediction by Semantic Evolution. In: ICC 2019-2019 IEEE International Conference on Communications (ICC). IEEE; 2019. p. 1–6.
11. Li X, Du N, Li H, Li K, Gao J, Zhang A. A deep learning approach to link prediction in dynamic networks. In: Proceedings of the 2014 SIAM International conference on data mining. SIAM; 2014. p. 289–297.
12. Chen J, Lin X, Jia C, Li Y, Wu Y, Zheng H, et al. Generative dynamic link prediction. Chaos: An Interdisciplinary Journal of Nonlinear Science. 2019;29(12):123111.
13. Chen J, Zhang J, Xu X, Fu C, Zhang D, Zhang Q, et al. E-LSTM-D: A deep learning framework for dynamic network link prediction. IEEE Transactions on Systems, Man, and Cybernetics: Systems. 2019;51(6):3699–3712.

14. Benson AR, Abebe R, Schaub MT, Jadbabaie A, Kleinberg J. Simplicial closure and higher-order link prediction. Proceedings of the National Academy of Sciences. 2018;115(48):E11221–E11230.

15. Liu B, Yang R, Lü L. Higher-order link prediction via local information. Chaos 1 August 2023; 33 (8): 083108. 2023;.

16. Piaggesi S, Panisson A, Petri G. Effective Higher-order Link Prediction and Reconstruction from Simplicial Complex Embeddings. In: Learning on Graphs Conference. PMLR; 2022. p. 55–1.

17. Liu Y, Ma J, Li P. Neural predicting higher-order patterns in temporal networks. In: Proceedings of the ACM Web Conference 2022; 2022. p. 1340–1351.

18. Zou L, Wang A, Wang H. Memory Based Temporal Network Prediction. In: Complex Networks and Their Applications XI: Proceedings of The Eleventh International Conference on Complex Networks and their Applications: COMPLEX NETWORKS 2022—Volume 2. Springer; 2023. p. 661–673.

19. Cencetti G, Battiston F, Lepri B, Karsai M. Temporal properties of higher-order interactions in social networks. Scientific reports. 2021;11(1):7028.

20. Ceria A, Wang H. Temporal-topological properties of higher-order evolving networks. Scientific Reports. 2023;13(1):5885.

21. Gallo L, Lacasa L, Latora V, Battiston F. Higher-order correlations reveal complex memory in temporal hypergraphs. Nature Communications. 2024;15. doi:10.1038/s41467-024-48578-6.

22. Iacopini I, Karsai M, Barrat A. The temporal dynamics of group interactions in higher-order social networks. Nature Communications. 2024;15(1). doi:10.1038/s41467-024-50918-5.

23. Di Gaetano L, Battiston F, Starnini M. Percolation and Topological Properties of Temporal Higher-Order Networks. Phys Rev Lett. 2024;132:037401. doi:10.1103/PhysRevLett.132.037401.

24. Jung-Muller M, Ceria A, Wang H. Higher-Order Temporal Network Prediction. In: Cherifi H, Rocha LM, Cherifi C, Donduran M, editors. Complex Networks & Their Applications XII. Cham: Springer Nature Switzerland; 2024. p. 461–472.

25. Fournet J, Barrat A. Contact patterns among high school students. PloS one. 2014;9(9):e107878.

26. Mastrandrea R, Fournet J, Barrat A. Contact patterns in a high school: a comparison between data collected using wearable sensors, contact diaries and friendship surveys. PloS one. 2015;10(9):e0136497.

27. Stehlé J, Voirin N, Barrat A, Cattuto C, Isella L, Pinton JF, et al. High-resolution measurements of face-to-face contact patterns in a primary school. PloS one. 2011;6(8):e23176.

28. Génois M, Barrat A. Can co-location be used as a proxy for face-to-face contacts? EPJ Data Science. 2018;7(1):1–18.

29. Isella L, Stehlé J, Barrat A, Cattuto C, Pinton JF, Van den Broeck W. What's in a crowd? Analysis of face-to-face behavioral networks. Journal of theoretical biology. 2011;271(1):166–180.

30. Vanhems P, Barrat A, Cattuto C, Pinton JF, Khanafer N, Régis C, et al. Estimating potential infection transmission routes in hospital wards using wearable proximity sensors. PloS one. 2013;8(9):e73970.

31. Ceria A, Havlin S, Hanjalic A, Wang H. Topological–temporal properties of evolving networks. Journal of Complex Networks. 2022;10(5):cnac041.

32. Zhao K, Stehlé J, Bianconi G, Barrat A. Social network dynamics of face-to-face interactions. Phys Rev E. 2011;83:056109. doi:10.1103/PhysRevE.83.056109.

33. Stehlé J, Barrat A, Bianconi G. Dynamical and bursty interactions in social networks. Phys Rev E. 2010;81:035101. doi:10.1103/PhysRevE.81.035101.

34. Abella D, Birello P, Di Gaetano L, Ghivarello S, Sabhahit NG, Sirocchi C, et al. Unraveling higher-order dynamics in collaboration networks. arXiv preprint arXiv:230617521. 2023;.
